# Short-term real-time prediction of total number of reported COVID-19 cases and deaths in South Africa: a data driven approach

**DOI:** 10.1186/s12874-020-01165-x

**Published:** 2021-01-11

**Authors:** Tarylee Reddy, Ziv Shkedy, Charl Janse van Rensburg, Henry Mwambi, Pravesh Debba, Khangelani Zuma, Samuel Manda

**Affiliations:** 1 Biostatistics Research Unit, South African Medical Research Council, Cape Town, South Africa; 2 Censtat, Hasselt University, Hasselt, Belgium; 3 School of Mathematics, Statistics and Computer Science, University of KwaZulu Natal, Durban, South Africa; 4 Smart Places Cluster, Council for Scientific and Industrial Research, Pretoria, South Africa; 5 Human and Social Capabilities Research Division, Human Science Research Council, Pretoria, South Africa; 6 Department of Statistics, University of Pretoria, Pretoria, South Africa

**Keywords:** Phenomenological models, COVID-19, Prediction, Richards model, Logistic growth model

## Abstract

**Background:**

The rising burden of the ongoing COVID-19 epidemic in South Africa has motivated the application of modeling strategies to predict the COVID-19 cases and deaths. Reliable and accurate short and long-term forecasts of COVID-19 cases and deaths, both at the national and provincial level, are a key aspect of the strategy to handle the COVID-19 epidemic in the country.

**Methods:**

In this paper we apply the previously validated approach of phenomenological models, fitting several non-linear growth curves (Richards, 3 and 4 parameter logistic, Weibull and Gompertz), to produce short term forecasts of COVID-19 cases and deaths at the national level as well as the provincial level. Using publicly available daily reported cumulative case and death data up until 22 June 2020, we report 5, 10, 15, 20, 25 and 30-day ahead forecasts of cumulative cases and deaths. All predictions are compared to the actual observed values in the forecasting period.

**Results:**

We observed that all models for cases provided accurate and similar short-term forecasts for a period of 5 days ahead at the national level, and that the three and four parameter logistic growth models provided more accurate forecasts than that obtained from the Richards model 10 days ahead. However, beyond 10 days all models underestimated the cumulative cases. Our forecasts across the models predict an additional 23,551–26,702 cases in 5 days and an additional 47,449–57,358 cases in 10 days. While the three parameter logistic growth model provided the most accurate forecasts of cumulative deaths within the 10 day period, the Gompertz model was able to better capture the changes in cumulative deaths beyond this period. Our forecasts across the models predict an additional 145–437 COVID-19 deaths in 5 days and an additional 243–947 deaths in 10 days.

**Conclusions:**

By comparing both the predictions of deaths and cases to the observed data in the forecasting period, we found that this modeling approach provides reliable and accurate forecasts for a maximum period of 10 days ahead.

**Supplementary Information:**

The online version contains supplementary material available at 10.1186/s12874-020-01165-x.

## Background

Coronaviruses are a large family of viruses which may cause respiratory infections ranging from the common cold to more severe diseases such as Middle East Respiratory Syndrome (MERS) and Severe Acute Respiratory Syndrome (SARS). The ongoing outbreak of the novel coronavirus (SARS-CoV-2) was first detected on 31 December 2019 in Wuhan, China. In the past 6 months the virus has rapidly spread to all regions with a total of 9,347,168 confirmed cases and 478,888 deaths as of 22 June 2020 [1].

The first COVID-19 case was reported in South Africa on 5 March 2020. By 22 June, 2020, South Africa had the highest burden of COVID-19 cases in the African region with 101,590 reported cases and 1991 confirmed COVID-19 related deaths. The South African government declared a national state of disaster on 15 March 2020 and commenced a state of lockdown from 26 March 2020 in an effort to reduce COVID-19 transmission in the country [2]. During this period all international and inter-provincial borders were closed, as well as all schools and several economic sectors in the country. In addition to these changes, non-pharmaceutical interventions such as the mandatory use of fabric masks, contact tracing and community testing were implemented across the country. As of June 2020, the country adopted a COVID-19 risk-adjusted strategy with a phased re-opening of selected economic sectors and schools. Due to the unprecedented nature of the situation, the uncertainties about the disease and the need to make informed policy decisions, modelling has taken centre stage in supporting key policy discussions surrounding COVID-19 in South Africa [3]. To date, the models that have been applied to the South African COVID-19 outbreak have focused on understanding the potential effects of interventions and policies based on SEIR-type models. These models are a common epidemiological modelling technique that divides a population into several compartments according to infection status (Susceptible, Exposed, Infectious, and Removed). Based on assumptions about the disease process, public health policies, demographic and mixing patterns among individuals in the population a set of differential equations governing how individuals in the population transition from one compartment to another, are defined and solved. Although these models are useful in understanding the effect of different factors on the transmission process and possible intervention strategies, they are sensitive to the assumptions made and require a deep understanding of the disease being modelled. The South African National COVID-19 Modeling Consortium [4], for example, assumed the following in their SEIR model: 75% of infected individuals are asymptomatic, the time from onset to infectiousness is 4 days (2∙0–9∙0), a 5 day duration of infectiousness from onset of symptoms; a mean of 9 days (8∙0–17∙0) between the time from onset of symptoms to ICU admission. Based on these assumption and model structure, it was predicted (June 12, 2020) that the number of detected cases (assuming the current detection rate of June 12) was 185,000 (89,500 - 358,000) and 278,000 (132,000 - 535,000) for the 29th of June and the 6th of July 2020, respectively. The observed number of cases corresponding to these dates were 144,264 and 205,721, respectively.

An alternative modelling approach, which is more robust and notably simpler (as it is not necessarily required to make assumptions about the transmission process) is that of phenomenological models. These non-linear epidemiological models have previously been applied to model other disease outbreaks such as Ebola [5], Dengue [6], Zika virus [7] and, more recently, the COVID-19 pandemic. Specifically, Roosa and colleagues fitted the generalized logistic model, Richards model and a sub-epidemic model to the cumulative COVID-19 cases in the Hubei province of China and the rest of China (excluding the Hubei province) and produced a short-term forecast of 5, 10 and 15 days ahead [8]. The authors expanded on this work using the same modelling approach for the provinces of Guandong and Zheijang [9]. In recent analysis a similar approach was taken to estimate the key epidemic parameters for all 11 provinces in China as well as 9 selected countries [10]. All the aforementioned papers have only focused on modelling cumulative COVID-19 cases. It can be argued, however, that both COVID-19 cases as well as COVID-19 deaths are of key importance in modelling the burden of COVID-19.

In using phenomenological models careful consideration needs to be given to the predictions emanating from all of the models fitted as such models could be used to support interventions on containing an epidemic. Generally authors select models on one of two key phenomenological modelling approaches: model selection and model averaging [7]. The former consists of selection of the model with the best goodness of fit to the data (and predicting the number of cases and epidemiological parameters of interest based on the selected model) and the latter uses information from a collection of models fitted to the data for prediction and estimation. The latter approach is a robust method to handle model uncertainty, particularly when there are several models which provide similar fits to the observed data. In the current paper and in the context of COVID-19 in Sub Saharan Africa we advocate the use of a sensitivity analysis approach for short (and long) term prediction of the number of cases.

In this paper we present (1) South Africa’s COVID trajectory to the first 100,000 (22 June 2020) cases and (2) fit a series of non-linear growth models, calibrated to COVID-19 cumulative number of reported case data from 5 March 2020 to 22 June 2020. The models are used to produce short term predictions of the number of reported cases expected for a period of 30 days ahead. These forecasts are generated at the national level as well as at the provincial level for the three highest burden provinces (Western Cape, Gauteng, and Eastern Cape). In view of the strong dependence of the number of detected COVID-19 cases and the number of tests performed, as well as the testing algorithm applied to the population, we also focus on the modelling of COVID-19 deaths, which may provide more reliable insight into the burden of the disease in South Africa. The short-term forecasts (for a period of 30 days ahead) of COVID-19 related deaths at a national level (and for selected provinces) are also studied.

Modelling the COVID-19 outbreak in South Africa implies modelling a dataset which is updated daily, which could affect the selection and the associated performance of the model on daily basis. Thus, while the selection of the best fitting model to the data could be critical, we illustrate the inherent analytical predictive problem of choosing a single model for predicting the future number of cases. Our point of view is that in a country such as South Africa (and other countries in Sub-Saharan Africa), where there is uncertainty related to the true number of cases, a sensitivity analysis based on multiple models is necessary for both short and long-term prediction of the number of cases and epidemiological parameters of interest.

## Methods

### Data

Routine confirmation of cases of COVID-19 is based on amplification and detection of unique SARS CoV-2 viral nucleic acid sequences by real-time reverse-transcription polymerase chain reaction (rRTPCR), with confirmation by nucleic acid sequencing when necessary [11]. A daily record of newly diagnosed COVID-19 cases and deaths were extracted for the period 5 March 2020 to 22 June 2020, at national and provincial level from a publicly available data repository [12, 13]. Data from the first 110 days of the outbreak (until June 22, 2020) were used to fit the models.

### Statistical analysis

For the analysis presented in this paper, we follow the modelling approach presented by Roosa et al. [8, 9] and Sebrango et al. [7] and fit a set of nonlinear growth models to the total number of reported cases and deaths. We let *Y*(*t*) denote the cumulative number of cases (or deaths) at time *t* and *μ*(*t*) represent the expected number of reported cases at time *t*. For the purpose of this study, we considered five data driven non-linear growth models, namely; 3 parameter logistic; 4 parameter logistic; Gompertz and Weibull growth models, which are presented in Table 1.

**Table 1:** Model formulation for the nonlinear models fitted to the COVID-19 outbreak data. Note that Y(t) is the daily expected cumulative number of cases and Y(t) = μ(t) + ε(t)

Model	
Richards	$\mu (t)=\alpha \left(1+k\exp {\left(-\gamma \left(t-\eta \right)\right)}^{-\frac{1}{k}}\right)$
3 Parameter logistic	$\mu (t)=\frac{\alpha }{1+\mathit{\exp}\left(-\gamma \left(t-\eta \right)\right)}$
4 Parameter logistic	$\mu (t)=\beta +\frac{\alpha -\beta }{1+\mathit{\exp}\left(-\gamma \left(t-\eta \right)\right)}$
Gompertz	$\mu (t)={\alpha}_0+\left(\alpha -{\alpha}_0\right)\exp \left(-\exp \left(-\gamma \left(t-\eta \right)\right)\right)$
Weibull	$\mu (t)={\alpha}_0+\left(\alpha -{\alpha}_0\right)\exp \left(-\left(\frac{t}{\eta}\right)k\right)$

The advantage of using the above models is that their mean structure μ(t) can be parametrized in terms of the growth rate, the final size and the turning point of the outbreak.

For all the models, the parameter α denotes the final size of the epidemic (i.e., the total number of reported cases at the end of the epidemic), γ the per capita intrinsic growth rate of the infected population, k the exponent of the deviation from the standard logistic curve and η the turning point (i.e. the time in which the daily number of cases reach its peak and the half time of the outbreak). Specifically Wu et al. [14] state that when an epidemic follows an exponential growth at an early stage the Richards model may be more suitable and that when the growth rate slows down (after the turning point) logistic models may provide a better fit to the data.

In line with approach adopted by [8, 9], the unknown model parameters were estimated using non-linear least squares estimation. This is achieved by searching for the set of parameters that minimizes the sum of squared differences between the observed data and the corresponding model solution. All analysis was performed using R and SAS. For outcomes with a clear biphasic trajectory, piecewise forms of the growth curves were fitted. The optimal change point was chosen by iteratively comparing fit criteria of models with different change points, with the model with the smallest Akaike information criteria [15] selected. In provincial models, the first time point (day 1) refers to the date at which the first case was diagnosed in the specific province. To assess the accuracy of the models in predicting cases and deaths, we present the actual observed values in the forecasting period for both cases and deaths.

### Prediction intervals

For the analysis presented in this paper, our main interest is to use the available data from *t* = 1 to *t* = *T* and to forecast the total number of cases for the period *T* + 30 days ahead. We term the period 1 to *T* the estimation period and the period from *T* + 1 to *T* + 30 the forecast (prediction period). To construct prediction intervals (within and outside the estimation period) we applied a parametric bootstrap [16] approach which was previously used to quantify parameter uncertainty and construct confidence intervals in mathematical modeling studies [17]. In this method, multiple observations are repeatedly sampled from the best-fit model in order to quantify parameter(s) and prediction uncertainty by assuming that the time series follows a Poisson distribution centered on the mean at the time points *t*_*i*_.

## Results

**Fig. 1:**
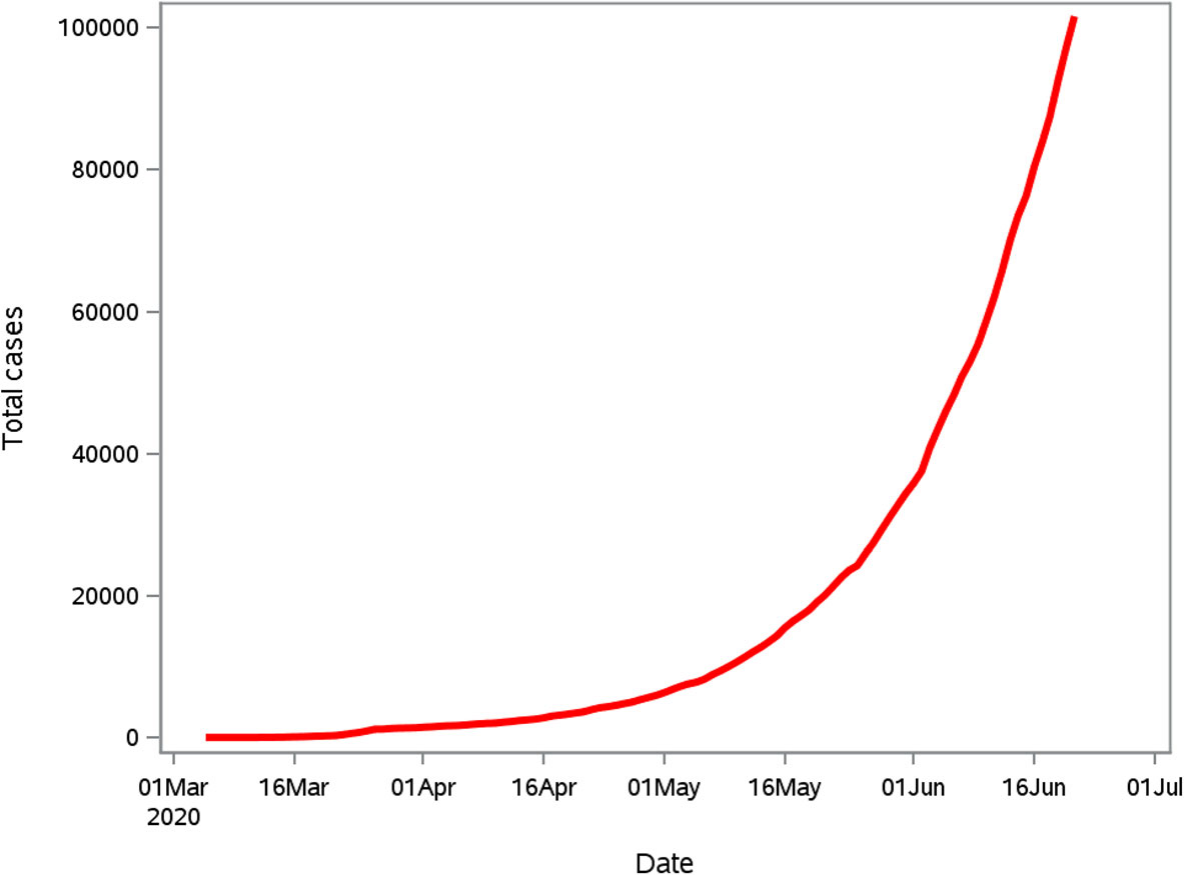
The cumulative COVID-19 cases for the period 5 March 2020 to 22 June 2020

The daily number of reported COVID-19 cases for the period 5 March 2020 to 22 June 2020 is presented in Fig. 1. The growth of COVID-19 in South Africa appears to be rapid until 27 March 2020 where a total of 243 daily new cases were observed, followed by a decline in the rate of new cases. From the 28 March 2020 to 11 April 2020 the daily increase in cases was consistently below 100. From May 2020 onwards a consistent increase more than 1000 cases per day were observed with larger increments in June.

**Fig. 2:**
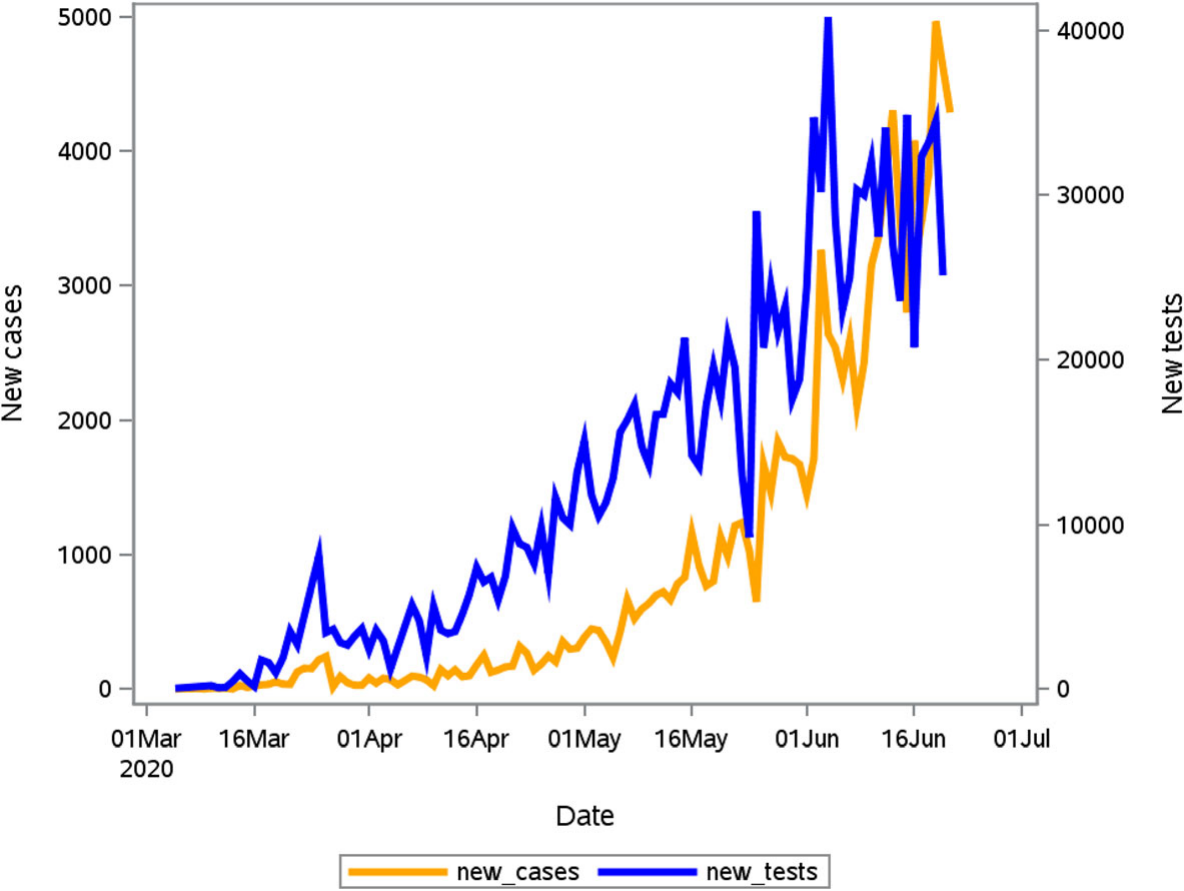
The relationship between daily COVID-19 tests and cases diagnosed for the period 5 March 2020 to 22 June 2020

The daily number of new reported COVID-19 cases and tests performed are presented in Fig. 2. To date, a total of 1,353,176 tests have been conducted, corresponding to a testing rate of 22.816 per 1000 population. There was a significant correlation between the number of cases detected and the number of tests performed daily (Rho = 0.7759, *p*-value< 0.001).

**Fig. 3:**
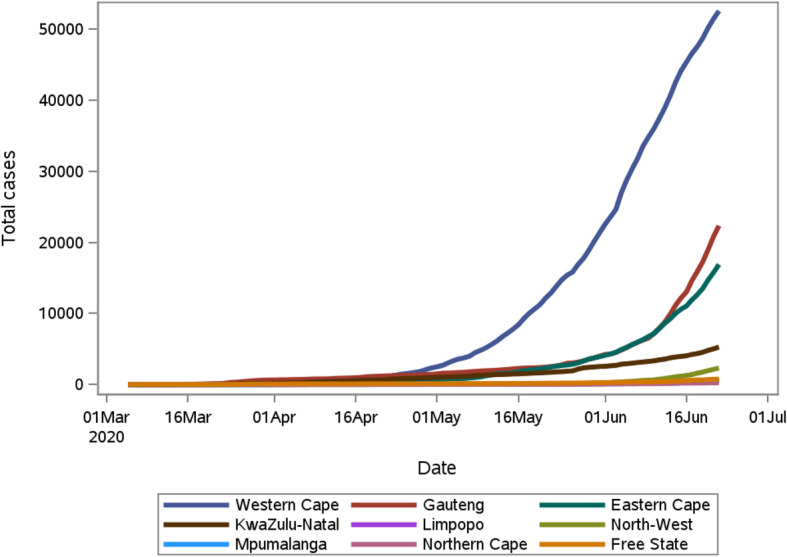
The cumulative COVID-19 cases in each of the nine provinces in South Africa for the period 5 March 2020 to 22 June 2020

The cumulative COVID-19 cases are depicted separately for each of South Africa’s nine provinces in Fig. 3, where a high degree of interprovincial heterogeneity is observed. As at 22 June 2020 the province with the highest number of cases is the Western Cape with 52554 cases, followed by Gauteng and Eastern Cape with 22341 and 16895 cases respectively.

**Fig. 4:**
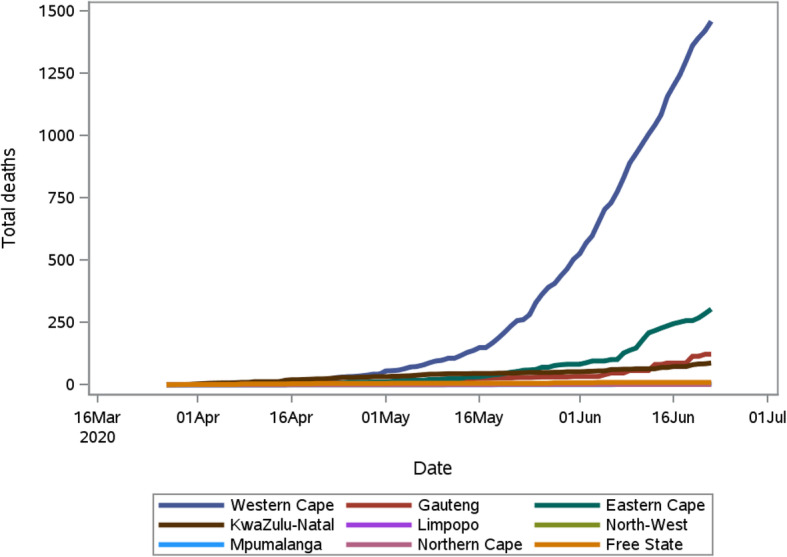
The cumulative COVID-19 deaths in each of the nine provinces in South Africa for the period 5 March 2020 to 22 June 2020

The total deaths reported from the 27 March to 22 June 2020 is presented in Fig. 4. In total 1991 COVID-19 related deaths were reported in this period with an overall case fatality rate of 1.96%. The first death was observed in the Western Cape (WC), followed by KwaZulu Natal (KZN), Free State (FS) and Gauteng Province (GP). Eastern Cape (EC) recorded their first death on the 16 April 2020. Initially WC contributed the most to the deaths as it was the epicentre. The other provinces curves’ exhibit patterns that are indicative of irregular reporting, as increases occurred in steps.

### Short-term prediction of the total number of reported COVID-19 cases - a national level analysis

**Fig. 5:**
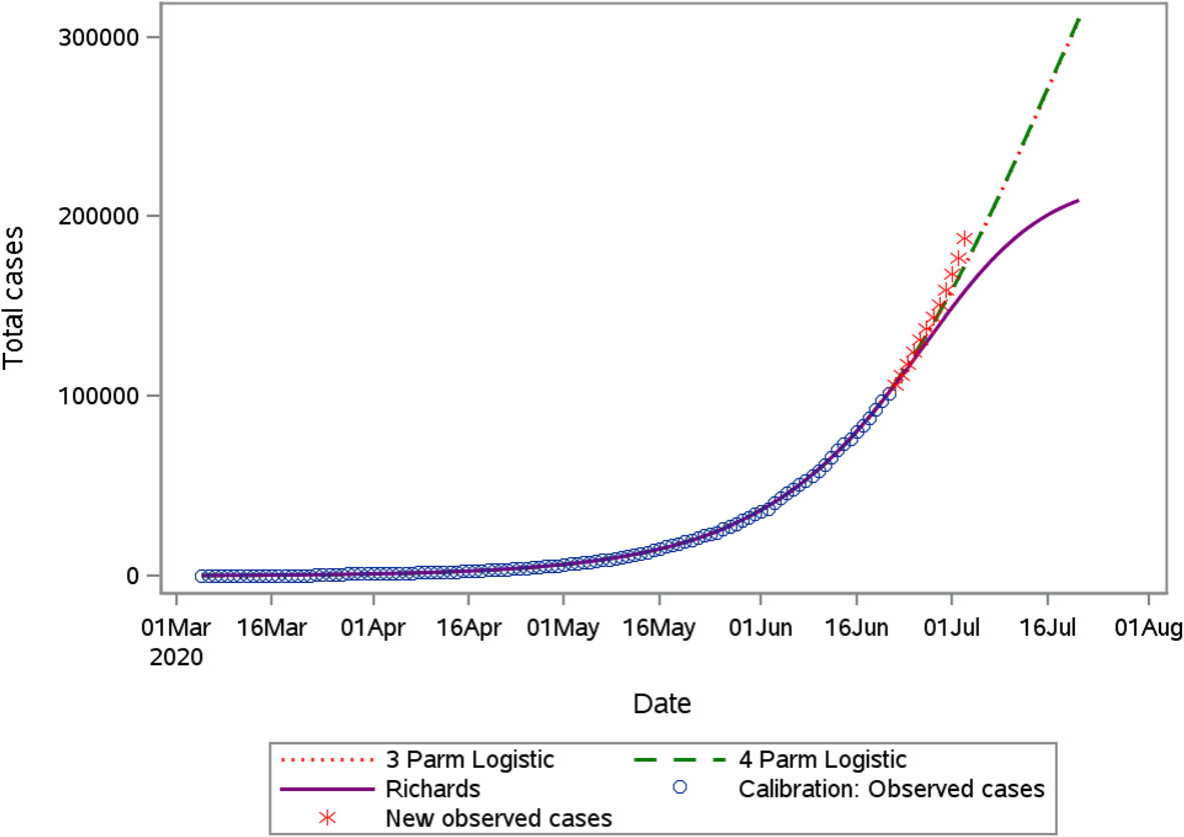
Predicted cumulative COVID-19 cases from the 3 Parameter logistic, 4 parameters logistic and the Richards model and observed cases

The models described in the previous section were all considered for modeling cumulative cases, with only models resulting in convergence further reported on in the tables. The Richards model, 3 and 4 parameter logistic models were fitted to the total number of reported COVID-19 cases at national level. The parameter estimates for the different models are presented in Supplementary Table 1. As mentioned in the previous section, our main interest is to produce a short term forecast for the number of reported cases and deaths. As depicted from the short-term forecasts for the three models fitted to cases (see Fig. 5, Table 2), all three models appear to fit the observed data (within the estimation period) well with the 3 parameter and 4 parameter logistic models providing very similar predictions over the 30-day ahead period. The AIC values are equal to 557, 559 and 555 for the 3PL, 4PL and Richards models, respectively, indicating that the Richards model is to be preferred considering in-sample predictions. However, it is clear from Fig. 5 that outside the estimation period, i.e., beyond June 22, 2020, the Richards model fits poorly. The predictive accuracy of the models to forecast the cumulative cases beyond the estimation period is presented graphically in Fig. 5, by superimposing the observed total number of reported cases (red asterisk) for the period 23 June to 4 July 2020. It is clear that all models underestimate the cumulative cases beyond 10 days and we observe that the Richards model yields substantially wider prediction intervals than the 3PL and 4PL models. The 5 days forecast (26 June 2020) obtained for 3PL model indicates that we can expect approximately a 30% increase in the cumulative COVID-19 cases in South Africa relative to 22 June 2020. For July 1, 2020, the 3PL predicts 158,859 (157047–160,633) cases and the observed number of cases is equal to 159,333. The prediction of Richards model for this period is 149,039 (143886–153,385). We notice that all the models underestimate the number of cases for a period of 30 days ahead, where the observed cumulative cases were 381,798.

**Table 2:** Short-term predictions of total number of reported cases at the national level under the 3 Parameter logistic, 4 parameter logistic and Richards model. Estimation period 05/03/2020–22/06/2020

	3 Parameter Logistic		4 Parameter Logistic		Richards model		Observed
Date	Prediction	Prediction interval	Prediction	Prediction interval	Prediction	Prediction interval	
26-Jun-20	128,257	127,305–129,275	128,292	127,071–129,025	125,141	123,394–126,557	124,590
1-Jul-20	158,859	157,074–160,633	158,948	156,884–160,560	149,039	143,886–153,385	159,333
6-Jul-20	193,359	190,007–196,599	193,543	189,877–196,681	170,681	159,866–180,740	205,721
11-Jul-20	230,852	225,263–236,388	231,182	225,077–236,880	188,091	170,432–206,750	264,184
17-Jul-20	270,005	261,460–278,480	270,539	261,003–279,896	200,661	176,633–229,649	324,221
22-Jul-20	309,224	297,266–321,792	310,020	296,347–323,957	208,985	179,833–247,952	381,798

The 3-parameter model provides accurate forecasts within the first 5 days. However, it is observed that beyond this point, observed values lie slightly outside of the prediction interval. This illustrates the need of a real time forecasting with daily calibration, particularly in the period approaching the peak where steep (and often random) increases are observed. Supplementary Figure 1 shows the predicted values and 95% CIs obtained from the three models for the 5 and 10 day time points. This figure illustrates that, while at each forecasting date a different model may provide an accurate forecast and prediction intervals which contain the true value, considering a joint prediction interval from all models contains the true value at all three dates. This interval is computed such that the overall lower bound is the minimum lower bound of the three model bootstrap prediction intervals. Similarly, the upper bound is defined as the maximum upper bound of the three model bootstrap prediction intervals.

### Short-term forecasts of the total number of reported COVID-19 cases – a province level analysis

As presented in Fig. 3, the outbreak in different provinces does not follow the same pattern and at the time that this analysis was conducted (June 22, 2020) three provinces (Western Cape, Eastern Cape and Gauteng) were responsible for 90.3% of the total diagnosed cases in South Africa. In this section we present a similar modeling approach implemented to a province-specific COVID-19 cumulative case trajectory for the three highest burden provinces. Of the four models fitted to the Western Cape, namely the 3 and 4 parameters logistic, Weibull and Richards model, the model which provided the best fit to the data is the 3 parameter logistic regression model. Due to the significantly slower growth rate of the outbreaks in the Eastern Cape and Gauteng from March, 2020 until 22 June 2020, piecewise growth models were fitted to capture this change point. The model which provided the best fit to the Eastern Cape data is the 3-parameter logistic model with a change point in the growth rate at day 80 (8 June 2020). Similarly, a piecewise 3 parameter logistic model was fitted to the Gauteng data with a change point at day 87 day (1 June 2020). The 30-day forecast of COVID-19 cases, in 5-day intervals, is presented for each province in Table 3. On 26 June 2020 (5-day forecast), the predicted number of cases were 57,481 (95% C. I 57135–58,197), 19,325 (18993–19,716) and 27,930 (27433–28,428) for Western Cape, Eastern Cape and Gauteng, respectively. The actual observed number of cases on 26 June 2020 were 57,941, 21,938 and 31,344 in the Western Cape, Eastern Cape, and Gauteng, respectively indicative of an underestimation of cases in Eastern Cape and Gauteng. Note that, similar to the total number of cases in the previous section, this underestimation is more pronounced for a 10 days (1 July 2020) forecast onward, where the observed cases in the Eastern Cape and Gauteng were 29,340 and 45,944, respectively.

**Table 3:** Short-term predictions of the total number of reported COVID-19 cases based on the 3PL model in Western Cape, Eastern Cape and Gauteng

	Western Cape			Eastern Cape			Gauteng		
Date	Prediction	Prediction Interval	Observed cases	Prediction	Prediction interval	Observed cases	Prediction	Prediction interval	Observed cases
26-Jun-20	57,481	57,135–58,197	57,941	19,325	18,993–19,716	21,938	27,930	27,433–28,428	31,344
1-Jul-20	62,129	61,733–63,044	64,377	20,952	20,437–21,541	29,340	32,623	31,783–33,566	45,944
6-Jul-20	65,668	65,228–66,756	70,938	21,516	20,904–22,210	38,081	34,927	33,770–36,240	66,891
11-Jul-20	68,259	67,768–69,501	77,336	21,693	21,040–22,439	48,232	35,905	34,614–37,415	93,044
17-Jul-20	70,399	69,867–71,785	84,254	21,751	21,083–22,519	58,860	36,294	34,942–37,904	123,408
22-Jul-20	71,592	71,020–73,069	87,847	21,764	21,092–22,536	67,818	36,445	35,067–38,100	144,582

### Short term forecasts of the number of COVID-19 related deaths

**Fig. 6:**
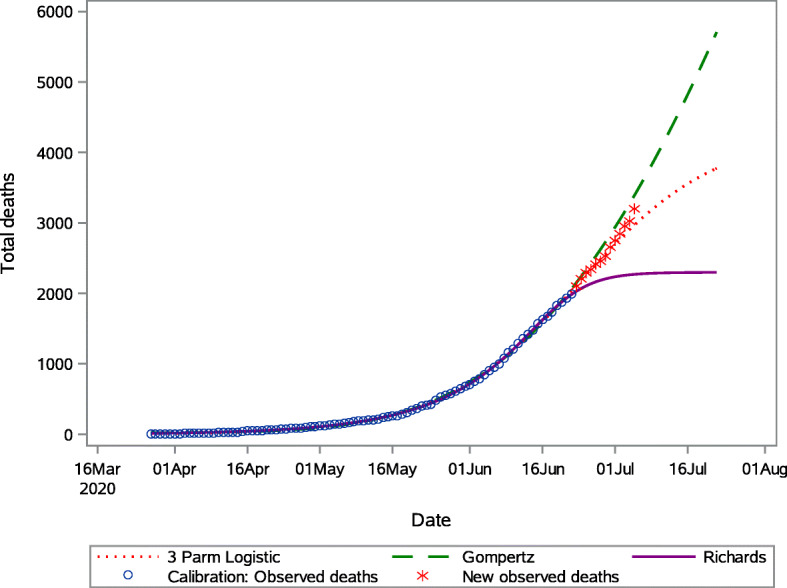
Predicted cumulative COVID-19 deaths in South Africa from the 3 Parameter logistic, Gompertz and Richards models

As previously mentioned, the total number of cases is highly correlated to the total number of tests conducted and therefore can be a misleading indicator to the outbreak progression. For that reason, modelling the total number of COVID-19 related deaths is of interest. We considered all the models described in Table 1 in the modeling of COVID-19 deaths. However, only the 3PL, Richards model and Gompertz model resulted in convergence and are subsequently reported on. The parameter estimates and fit criteria for each of the models fitted are presented in Supplementary Table A2. According to the AIC, within the estimation period, the Richards model provided the best fit to the cumulative COVID-19 deaths at the national level. As with the total number of cases, the Richards model seems to flatten out prematurely and therefore, although it fitted the data better according to the AIC, the 3PL forecasts are used in subsequent interpretation. The 30-day forecast of COVID-19 deaths, at 5-day intervals, is presented in Table 4. The predicted number of deaths on the 26 June 2020 (5 day forecast) is 2336 (2219.4–2457.2), and the total deaths is expected to be 2700 (2534.7–2883.6) as at 1 July 2020. The 30-day forecast is subject to a higher degree of uncertainty with 3775 (3346.3–4435.1) deaths prediction. We notice that the 3PL model provides smaller predictions compared to the Gompertz model but higher predictions compared to the Richards models. Figure 6 shows the observed deaths and the predictions obtained from the fitted models, as well as the superimposed reported deaths for the period 23 June to 4 July 2020. It is clear from this graph that the model which most closely captures the trajectory of COVID-19 related deaths in SA, outside the estimation period, is the 3PL model.

**Table 4:** Short-term predictions of the total of COVID-19 related deaths at the national level obtained for the 3 Parameter logistic, Gompertz and Richards model

	3 Parameter logistic		Gompertz		Richards		Observed deaths
Date	Prediction	Prediction Interval	Prediction	Prediction Interval	Prediction	Prediction Interval	
26-Jun-20	2335.60	(2219.46–2457.20)	2427.55	(2307.86–2548.39)	2135.91	(2026.155–2272.12)	2340
1-Jul-20	2699.07	(2534.78–2883.60)	2937.94	(2762.25–3120.19)	2234.63	(2076.82–2461.46)	2749
6-Jul-20	3030.89	(2808.62–3309.00)	3507.83	(3259.49–3783.43)	2274.72	(2084.222–2593.48)	3310
11-Jul-20	3317.85	(3029.44–3715.10)	4135.71	(3768.47–4560.75)	2289.68	(2085.09–2677.04)	3971
17-Jul-20	3595.95	(3230.76–4142.80)	4961.96	(4419.59–5621.05)	2295.63	(2085.252–2724.29)	4804
22-Jul-20	3774.63	(3346.30–4435.10)	5706.81	(4961.87–6607.89)	2297.18	(2085.271–2745.95)	5940

Due to the low COVID-19 death rate in South Africa, the distribution of the cumulative deaths at the provincial level posed a greater challenge in terms of modelling, and we were only able to fit models to the Western Cape COVID-19 deaths.

**Fig. 7:**
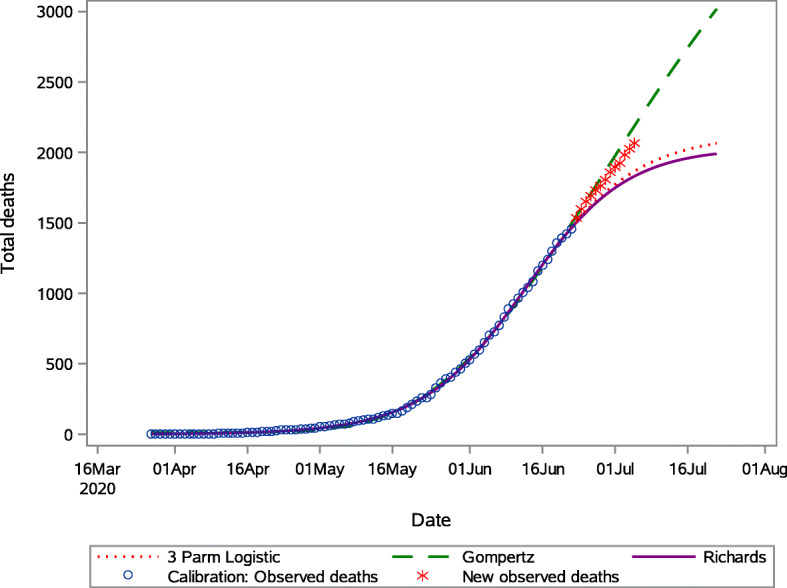
Predicted cumulative COVID-19 deaths from the 3 Parameter logistic, Gompertz and the Richards model for Western Cape

The model predictions with observed deaths for Western Cape is presented in Fig. 7. The 3PL model and the Richards model predictions are close and fit the observed data the best. Figure 7 indicates that the forecasts of the Gompertz model are closest to observed values beyond 22 June 2020. We can see that the trajectory of the Gompertz forecasts beyond the 30 day forecast window will overestimate the observed cases, assuming the actual deaths continue its observed path (Table 5).

**Table 5:** Short-term predictions of the total of COVID-19 related deaths for the Western Cape Province

	3 Parameter logistic		Gompertz		Richards		Observed deaths
Date	Prediction	Prediction Interval	Prediction	Prediction Interval	Prediction	Prediction Interval	
26-Jun-20	1619.6	(1531.5–1707.9)	1708.3	(1612.6–1797.9)	1608.4	(1509.7–1707.6)	1692
1-Jul-20	1772.6	(1665.2–1884.5)	1975.5	(1848–2104.1)	1748.7	(1610.7–1897)	1896
6-Jul-20	1886.1	(1754.5–2026.8)	2239.9	(2074–2417.4)	1848.2	(1666.9–2063.9)	2101
11-Jul-20	1966.3	(1815.9–2129.3)	2496.6	(2270.7–2733.8)	1915.2	(1695.5–2188)	2333
17-Jul-20	2029.6	(1864.5–2217.6)	2789.4	(2496.5–3119.8)	1965.3	(1711.9–2302.8)	2550
22-Jul-20	2063.2	(1887.5–2265.7)	3017.6	(2654–3430.5)	1990.6	(1717.4–2381.4)	2752

## Discussion

In view of the existing healthcare challenges faced by South Africa, reliable and accurate short-term forecasts of COVID-19 cases and deaths are critical to ensure optimal resource allocation and should be a key aspect of the strategy to handle the COVID-19 epidemic in the country. This study modelled COVID-19 cases and deaths using publicly available data from 5 March 2020 to 22 June 2020. Five data-driven nonlinear growth models, namely the Richards; 3 parameter logistic; 4 parameter logistic; Gompertz and Weibull were considered.

We observed that models for cases and deaths provide robust and accurate short-term forecasts for a period of 10 days ahead at the national level. However, given the rapidly changing growth rate as the country approaches the COVID-19 peak, as well as the changes to COVID-19 regulations and the reopening of the economy, it is crucial that these models are fitted daily as new data becomes available and that forecasts are updated and reported accordingly on a daily basis. In addition, we observed difficulty in fitting models at the provincial level, particularly for provinces which are relatively “early” in their COVID-19 outbreak. There were also convergence problems encountered when fitting the five models, resulting in us only reporting on the three specific models which converged to cumulative cases and deaths. It is important to note that all these models have limitations and may only be applicable in certain stages of the outbreak, or when enough data are available for stable estimation of parameters.

Moreover, based on the results presented in this paper we recommend not to base the forecasting on a single model or to apply a modeling averaging technique to the results obtained from the different models. Rather we propose to use different models as a tool to estimate a realistic uncertainty interval of the predictions. As we have observed, models that have the best goodness to fit within the estimation period can predict poorly beyond the estimation period (which is the primary interest during the outbreak). At each forecasting date, a different model provides an accurate forecast and prediction intervals. However, if we use the prediction intervals obtained from all models, we will cover the observed values at both dates. Once again, we re-emphasize that we do not recommend using our modeling approach beyond a forecasting for 10 days ahead.

Although the time for South Africa to reach 100,000 cumulative cases of COVID-19 was approximately 110 days since the first reported case, our forecasts reveal that the country should be prepared for an additional 47,449–57,358 cases within the next 10 days. This reinforces the need for the public to adhere to all the non-pharmaceutical interventions that have been emphasized, such as social distancing, washing of hands and the wearing of masks.

A strength of the analysis presented in this paper is that it was conducted using readily accessible, publicly available data which is updated in real time. In addition, the statistical methods applied are relatively simple, intuitive and are not dependent on any assumptions regarding COVID-19 transmission dynamics which may be unknown (or the current knowledge can be misleading). Other researchers who modelled the COVID-19 outbreak in South Africa using the SEIR approach [4], predicted that the number of detected cases (assuming the detection rate of June 12) was 185,000 (89,500 - 358,000) and 278,000 (132,000 - 535,000) for the 29th of June and the 6th of July 2020, respectively. The observed number of cases corresponding to these dates were 144,264 and 205,721, respectively. These prediction intervals were substantially wider and further from the true observed values than those produced by our modelling approach. This advocates that a data driven approach, while unreliable beyond 10 days ahead, does provide more accurate forecasts in this period.

A limitation, however, is that we can only predict laboratory confirmed *diagnosed* COVID-19 cases and reported deaths attributed to COVID-19. Therefore, it is possible that the true burden of COVID-19 in the country, considering asymptomatic or pre-symptomatic undiagnosed cases may be much higher than that observed. In light of these limitations, the modeling of COVID-19 deaths is crucial to gain greater insight into the COVID-19 burden in South Africa. However, due to the low numbers of deaths up to 22 June, as well as the way in which the death data was reported as was seen in the step-wise patterns observed in some provinces, modelling deaths using phenomenological models requires caution. While these model-based predictions of COVID-19 deaths reveal that approximately 1500 new deaths can be expected by 22 July 2020, it is important to interpret these numbers cautiously in light of the evidence of a high number of excess deaths in the country [18].

Based on the analysis presented in this paper, a web-based platform (https://www.samrc.ac.za/content/covid-19-forecasts) was developed in which the observed number of cases and deaths, as well as short-term forecasts are presented. In this way policy makers and the general public can consult the website and get a reliable understanding, supported by evidence observed in the data, about the COVID-19 outbreak in South Africa. A detailed description of platform will be given in a future publication.

We have shown the usefulness of non-linear growth models to provide short term forecasts of COVID-19 cases and deaths in South Africa. We focused on the short-term prediction of cumulative COVID-19 cases and deaths, and while the estimates of the turning point of the outbreak and final size of the epidemic are presented in the appendix, these parameters are not interpreted in the results. The rationale behind this decision, which was exemplified even in the forecasting of cumulative cases and deaths beyond 14 days, is that the daily confirmed COVID-19 cases and deaths are rapidly changing, as are the reporting and testing guidelines in the country. An area of further work involves a comprehensive assessment of the models applied for long term prediction and internal validation of the model.

## Conclusions

This study found that the phenomenological modeling approach provides reliable and accurate forecasts of COVID-19 cases and deaths in South Africa for a maximum period of 10 days ahead. In view of the rapidly changing growth rate as the country approaches the COVID-19 peak, as well as the changes to COVID-19 regulations and the reopening of the economy, we recommend that these models are fitted daily to the latest COVID-19 cumulative cases and deaths data.

## Supplementary Information

**Additional file 1.**

## Data Availability

The dataset used and/or analyzed during the current study are available free to the public from the website https://github.com/dsfsi/covid19za

## Abbreviations

AIC: Akaike Information criteria
3PL: Three parameter logistic
4PL: Four parameter logistic
ICU: Intensive care unit
EC: Eastern Cape
WC: Western Cape
KZN: KwaZulu Natal
FS: Free State
GP: Gauteng Province
SEIR: Susceptible, Exposed, Infectious, and Removed
