# Supplementary material for: Short-term real-time prediction of total number of reported COVID-19 cases and deaths in South Africa: a data driven approach
Source: BMC Med Res Methodol. 2021 Jan 11;21:15. doi: 10.1186/s12874-020-01165-x (PMC7797353; doi:10.1186/s12874-020-01165-x)
Supplement: Supplementary file 1 — Additional file 1. [file 12874_2020_1165_MOESM1_ESM.docx]

Supplementary information

Supplementary Tables

Table A1: Parameter estimates and Fit criteria for the three models fitted to cumulative COVID-19 cases in South Africa

|  | **3PL** | | **4PL** | | **Richards** | |
| --- | --- | --- | --- | --- | --- | --- |
| **Parameter** | **Estimate** | **Standard error** | **Estimate** | **Standard error** | **Estimate** | **Standard error** |
| alpha | 543214 | 31796 | 547056 | 40321 | 221501 | 38424 |
| gamma | 0.0581 | 0.0004 | 0.0581 | 0.0005 | 0.1145 | 0.0206 |
| eta | 134.2000 | 1.3581 | 134.4000 | 1.7442 | 114.7000 | 3.2539 |
| beta |  |  | -10.9285 | 68.1619 |  |  |
| k |  |  |  |  | 2.0401 | 0.3804 |
| **Fit Criteria** |  |  |  |  |  |  |
| AIC | 557.16 |  | 559.15 |  | 555.04 |  |
|  |  |  |  |  |  |  |

Table A2: Parameter estimates and Fit criteria for the three models fitted to cumulative COVID-19 deaths in South Africa

|  | **3PL** | | **Gompertz** | | | **Richards** | | |
| --- | --- | --- | --- | --- | --- | --- | --- | --- |
| **Parameter** | **Estimate** | **Standard error** | **Estimate** | **Standard error** | **Estimate** | | **Standard error** |  |
| alpha | 4279 | 168 | 34567 | 10480 | 2298 | | 62 |  |
| gamma | 0.07034 | 0.000961 | 0.01499 | 0.001318 | 0.208 | | 0.018067 |  |
| eta | 89.39 | 0.9937 | 157.38 | 13.1187 | 80.04 | | 0.2543 |  |
| beta |  |  | 21.4600 | 5.2200 |  | |  |  |
| k |  |  |  |  | 3.3970 | | 0.3180 |  |
| **Fit Criteria** |  |  |  |  |  | |  |  |
| AIC | 749.4 |  | 801.9 |  | 675.3 | |  |  |

Supplementary figure 1: Predicted cumulative COVID-19 cases from all models for 26/06/2020 and 01/07/2020. The solid horizontal lines are the observed number of cases (124590 and 159333 for 26/06 and 01/07, respectively). The red dashed lines are the upper and lower uncertainty intervals obtained from all fitted models.
